# Protective Effects of Aryl Hydrocarbon Receptor Signaling in Celiac Disease Mucosa and in Poly I:C-Induced Small Intestinal Atrophy Mouse Model

**DOI:** 10.3389/fimmu.2019.00091

**Published:** 2019-02-04

**Authors:** Vincenzo Dinallo, Irene Marafini, Davide Di Fusco, Antonio Di Grazia, Federica Laudisi, Rami Dwairi, Omero A. Paoluzi, Giovanni Monteleone, Ivan Monteleone

**Affiliations:** ^1^Department of Systems Medicine, Gastroenterology, University of Tor Vergata, Rome, Italy; ^2^Department of Biomedicine and Prevention, University of Tor Vergata, Rome, Italy

**Keywords:** aryl hydrocarbon receptor (AhR), celiac disease, intestinal atrophy, inflammation, IFN-gamma

## Abstract

Aryl hydrocarbon receptor (AhR), a transcription factor activated by a large number of natural and synthetic agents, modulates the activity of immune cells in the gut and represents an important link between the environment and immune-mediated pathologies. In this study, we investigated the role of AhR in celiac disease (CD), a gluten-driven enteropathy. AhR expression was evaluated in intestinal biopsies taken from patients with CD and controls by real-time polymerase chain reaction (PCR), immunohistochemistry and flow cytometry. AhR was also analyzed in *ex vivo* organ cultures of duodenal biopsies taken from inactive CD patients incubated in presence or absence of peptic-tryptic digest of gliadin. IFN-γ, TNF-α, granzyme B, and perforin expression was evaluated in anti-CD3/CD28-activated intestinal lamina propria mononuclear cells (LPMC) and intestinal intra-epithelial cells (IEL) of active CD patients cultured in the presence or absence of the AhR agonist 6-formylindolo(3, 2-b)carbazole (Ficz). Finally, the protective role of AhR was evaluated in a mouse model of poly I:C-driven small intestine damage. AhR RNA transcripts were reduced in active CD samples as compared to inactive CD and normal controls. Flow cytometry confirmed such results and showed a reduction of AhR in both IEL and LPMC of active CD patients. The addition of a peptic-tryptic digest of gliadin to *ex vivo* organ cultures of duodenal biopsies taken from inactive CD patients reduced AhR expression. Treatment of CD IEL and LPMC with Ficz reduced the levels of inflammatory cytokines, granzyme B and perforin. Mice injected with Ficz were protected against poly I:C-induced intestinal lesions. Our findings suggest that defective AhR-driven signals could contribute to amplify pathogenic responses in the gut of CD patients.

## Introduction

Celiac disease (CD) is a chronic enteropathy triggered by dietary gluten present in wheat, barley, and rye (1, 2). In genetically predisposed individuals, ingestion of gluten associates with activation of both innate and adaptive immune responses and production of elevated levels of inflammatory cytokines, with the downstream effect of causing villous atrophy and crypt hyperplasia (3). Several HLA-DQ2- and HLA-DQ8-restricted gluten peptides can promote T helper (Th)-type 1 polarization with consequent synthesis of interferon (IFN)-γ and interleukin (IL)-21 by CD4+ T cells (4–7). Gluten can also stimulate innate immune cells and epithelial cells to produce IL-15, which in turn activates cytotoxic cells, such as natural killer (NK) cells and CD8+ T lymphocytes (6). These activated cytotoxic cells bind to specific stress ligands on enterocytes, with the ultimate result of epithelial cell killing and villous atrophy (8). There is also evidence that CD-associated pathogenic response can be sustained by a defective activity of counter-regulatory mechanisms. For example, Benahmed et al. demonstrated that in active CD high levels of IL-15 impair Smad3-dependent TGF-β signaling, thus sustaining intestinal inflammation (9). Along the same line is the observation that elevated levels of Smad7, an intracellular inhibitor of TGF-β pathway, sustain the production of inflammatory cytokines in refractory CD (10). Moreover, it has been demonstrated that IL-10 polymorphisms, which lead to reduced production of IL-10, are associated with an early-onset of CD and a more clinically severe form of intestinal lesions (11).

Aryl hydrocarbon receptor (AhR) is a member of the Pern-Arnt-Sim (PAS) superfamily of transcription factors that are involved in sensing environmental signals such as changes in the circadian rhythm, oxygen tension, or redox potential (12). Following activation, AhR translocates from the cytoplasm to the nucleus where it controls the transcription of a wide variety of target genes (12). Initially known as the mediator of the toxic effects of dioxins, AhR can bind and be activated by multiple dietary compounds, derivatives of tryptophan, such as 6-formylindolo(3, 2-b) carbazole (Ficz) and commensal flora (13, 14). During the last years many studies have shown that AhR signaling regulates important immune reactions, both in health and diseases (15). For instance, AhR controls the differentiation, activation, and proliferation of many immune cells (15) and promotes the expansion of Th17 cells (16, 17). At the same time, AhR activation triggers anti-inflammatory pathways, which are dependent on induction of regulatory T cells (Tregs) (18), differentiation of IL-10 expressing T regulatory type 1 cells (19) and induction of tolerogenic dendritic cells (20, 21). In this context, we have shown that activation of AhR in intestinal mucosal cells stimulates the production of IL-22 and down-regulates the production of inflammatory cytokines, such as IFN-γ and TNF-α, thereby attenuating experimental colitis in mice (22). In this study, we investigated the expression and role of AhR in CD.

Collectively, our data show that AhR expression is down-regulated in inflamed mucosa of CD patients. Moreover, we show that AhR activation with Ficz reduces production of pro-inflammatory cytokines and cytotoxic factors in CD mucosal cells and attenuates small intestinal lesions in mice.

## Materials and Methods

### Patients and Samples

Duodenal biopsies were taken from 23 patients with active CD at the time of diagnosis (median age 29, range 22–40), 21 patients with inactive CD (median age 34, range 25–47) on a gluten-free diet and 18 normal controls (median age 39, range 30–53). All patients with active CD were on a gluten containing diet, were positive for both IgA anti-endomysium (EMA) and IgA anti-tissue transglutaminase 2 (TG2) and had villous atrophy on histological examination. Inactive CD patients were on a gluten-free diet for at least 2 years and were EMA and anti-TG2 negative and none of them had villous atrophy on histological examination. Control group included duodenal biopsies of patients who underwent upper endoscopy for gastrointestinal symptoms, had no macroscopic/microscopic alteration, and were EMA and anti-TG2 negative. Each patient who took part in the study gave written informed consent and the independent local Ethics Committee of the University hospital of Tor Vergata approved the study protocol.

### Murine Model of Small Intestinal Atrophy

All reagents were from Sigma-Aldrich (Milan, Italy) unless specified. Eight week-old female C57BL/6J wild type (WT) and AhR knockout (KO) mice (B6.129-*Ahr*^*tm*1*Bra*^/J, Jackson Laboratories, Bar Harbor, ME) were given intra-peritoneally polyinosinic:polycytidylic acid (poly I:C) (15 μg/g) dissolved in phosphate buffered saline (PBS) or PBS only (controls) and sacrificed 12 h later through cervical dislocation. One hour after poly I:C administration, mice were given Ficz (1 μg/mouse). This dose was selected based upon our previous studies, which documented activation of AhR in the intestinal mucosa following i.p. administration of the compound (22). Small intestine was harvested for histology, RNA extraction, LPMC, and IEL isolation. The murine experiments were approved by the local Institutional Animal Care and Use Committee of the University of Tor Vergata.

### Cell Isolation and Culture

Human LPMC and IEL were isolated as previously described with minor modifications (23). Briefly, biopsies taken from controls, inactive CD patients and active CD patients were freed of mucus with dithiothreitol. Cells were treated with ethylenediminetetracetic acid to separate epithelial cells from the lamina propria. EDTA suspension was then centrifuged to obtain IEL. The remaining tissue was digested with liberase (0.2 mg/ml; Roche, Mannheim, Germany) and DNase I (0.2 mg/ml; Roche). IEL and LPMC were resuspended (1 × 10^6^/ml) in RPMI-1640 supplemented with 10% fetal bovine serum, penicillin (100 μg/ml), streptomycin (100 μg/ml), and gentamycin (50 μg/ml; Lonza, Milan, Italy). Cells were either freshly stained for flow cytometry analysis or re-incubated with activating anti-CD3/CD28 beads (Miltenyi Biotec, Calderara di Reno, Italy), and 1 h after stimulated with Ficz (final concentration, 200 nmol/L; Alexis, Milan, Italy) for 48 h and then analyzed by flow cytometry. In each experiment, cell viability was evaluated using flow-cytometry. Phorbol myristate acetate (PMA, 10 ng/ml), ionomycin (1 mg/ml), and brefeldin A (10 mg/ml; eBioscience, San Diego, CA) were added to the cultures in the last 3 h in order to evaluate cytokine production.

### *Ex vivo* Organ Cultures

Freshly obtained duodenal biopsies of inactive CD patients were placed on sterile filters (EMD Millipore, Milan, Italy) in an organ culture chamber at 37°C in a 5% CO_2_/95%O_2_ atmosphere in AQIX medium (Liquid life, London, United Kingdom). Biopsies obtained from inactive CD patients were cultured with or without a peptic–tryptic digest of gliadin (PT, 1 mg/ml) and Ficz (final concentration, 200 nmol/L; Alexis, Milan, Italy) for 24 h. AhR, TNF-α, and IFN-γ mRNA relative expression was evaluated by real time PCR.

### Immunohistochemistry

Immunohistochemistry was performed on archival formalin-fixed paraffin-embedded duodenal sections of 4 patients with active CD and 4 controls. The sections were deparaffinized and dehydrated through xylene and ethanol and the antigen retrieval was performed in citrate buffer (pH 6.0) for 20 min in a microwave. Immunohistochemical staining was performed using a mouse monoclonal antibody directed against human AhR (ab2770, 1:150 final dilution; Abcam, Cambridge, MA, USA) at room temperature for 1 h followed by a biotin-free HRP-polymer detection technology with 3,3'diaminobenzidine (DAB) as a chromogen (UltraVision kit, Lab Vision, Fremont, CA, USA). The sections were counterstained with haematoxylin, dehydrated, and mounted. Isotype control IgG-stained sections were prepared under identical immunohistochemical conditions as described above, replacing the primary AhR antibody with a purified control isotype (R&D Systems).

### RNA Extraction, Complementary DNA Preparation, and Real-Time Polymerase Chain Reaction

RNA isolation, reverse transcription of the RNA, and real-time PCR were carried out as previously described. RNA was extracted by using TRIzol reagent according to the manufacturer's instructions (Invitrogen, Carlsbad, CA, USA). A constant amount of RNA (1 μg/sample) was reverse transcribed into complementary DNA, and this was amplified using the following conditions: denaturation for 1 min at 95°C; annealing for 30 s at 58°C for human and mouse AhR, human IFN-γ, and granzyme B, and mouse TNF-α, at 62°C for human TNF-α, at 60°C for human and mouse β-actin, mouse IFN-γ, perforin, granzyme B, and human perforin, followed by 30 s of extension at 72°C. All real-time PCR data were normalized to β-actin. Human primer sequences were as follows: AhR forward 5′-TACAGAGTTGGACCGTTTGG-3′, reverse 5′-GCCTCCGTTTCTTTCAGTAG-3′; IFN-γ forward 5′-TGGAGACCATCAAGGAAGAC-3′, reverse 5′-GCGTTGGACATTCAAGTCAG-3′; TNF-α forward 5′-AGGCGGTGCTTGTTCCTCAG-3′, reverse 5′-GGCTACAGGCTTGTCACTCG-3′; Granzyme B forward 5′-CAGTACCATTGAGTTGTGCG-3′, reverse 5′-GCCATTGTTTCGTCCATAGG-3′; Perforin forward 5′-CCAACTTTGCAGCCCAGAAG-3′, reverse 5′-GGAGATAAGCCTGAGGTAGG-3′; β-actin forward 5′-AAGATGACCCAGATCATGTTTGAGACC-3′, reverse 5′-AGCCAGTCCAGACGCAGGAT-3′. Mouse primer sequences were as follows: AhR forward 5′-GAGCACAAATCAGAGACTGG-3′, reverse 5′-TGGAGGAAGCATAGAAGACC-3′; IFN-γ forward 5′-CAATGAACGCTACACACTGC-3′, reverse 5′-CCACATCTATGCCACTTGAG-3′; TNF-α forward 5′-ACCCTCACACTCAGATCATC-3′, reverse 5′-GAGTAGACAAGGTACAACCC-3′; Granzyme B forward 5′-CTGCTAAAGCTGAAGAGTAAGG-3′, reverse 5′-ACCTCTTGTAGCGTGTTTGAG-3′; Perforin forward 5′-CCACTCCAAGGTAGCCAAT-3′, reverse 5′-GGAGATGAGCCTGTGGTAAG-3′; β-actin forward 5′-AAGATGACCCAGATCATGTTTGAGACC-3′, reverse 5′-AGCCAGTCCAGACGCAGGAT-3′. Gene expression was calculated using the ΔΔCt algorithm.

### Flow Cytometry

Cells were immunostained with the following monoclonal anti-human antibodies: APC-H7 anti-CD45, pacific blue anti-CD3, PeCy7 anti-CD8, V450 anti-CD56, APC anti-IFN-γ, (BD Bioscience, San Jose, CA), APC Alexa Fluor 700 anti-CD4 (Beckman Coulter, Milan, Italy), Percp Cy 5.5 anti-TNF-α (eBioscience, Milan, Italy), APC anti-perforin, and PE anti-granzyme B (Invitrogen). Cells were immunostained with the following anti-mouse antibodies: APC-Cy7 anti-CD45, FITC anti-IFN-γ (BD Bioscience), PE anti-TNF-α, APC anti-perforin, and Pecy7 anti-granzyme B (eBioscience). In all experiments, appropriate isotype control IgGs (Becton Dickinson and eBioscience) and fluorescence minus one controls were used. All antibodies were used at 1:100 final dilution. For intracellular immunostaining, cells were fixed and permeabilized using staining buffer set and permeabilization buffer (both from eBioscience) according to the manufacturer's instruction. Cells were analyzed by flow cytometry (Gallios, Beckman Coulter, Indianapolis, IN).

### Statistical Analysis

Differences between groups were compared using Student's *t*-test. All analyses were performed using GraphPad Prism version 5.00 software for Windows (GraphPad Software, San Diego California, USA, www.graphpad.com).

## Results

### AhR Expression Is Down-Regulated in Active Celiac Disease

**Figure 1:**
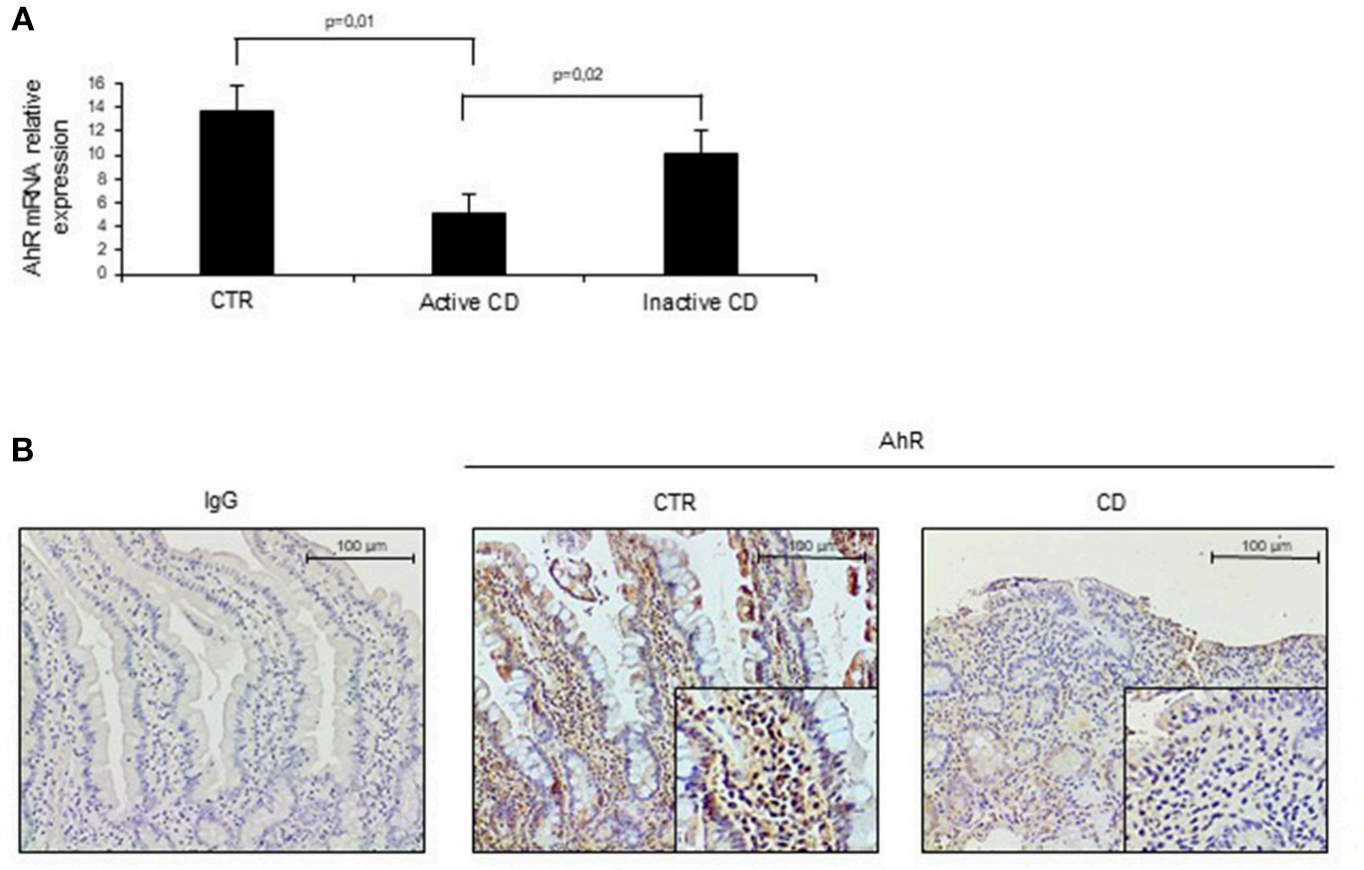
Aryl (AhR) expression is down-regulated in active celiac disease (CD). **(A)** Total RNA was extracted by duodenal biopsies of 13 normal controls (CTR), 12 active CD patients and 7 inactive CD patients and analyzed by real time PCR for AhR expression. **(B)** Representative immunohistochemical images showing AhR-positive cells in duodenal sections of normal controls (CTR) and active CD patients. Staining with isotype control IgG is also shown. These images are representative of 4 separate experiments in which similar results were obtained.

**Figure 2:**
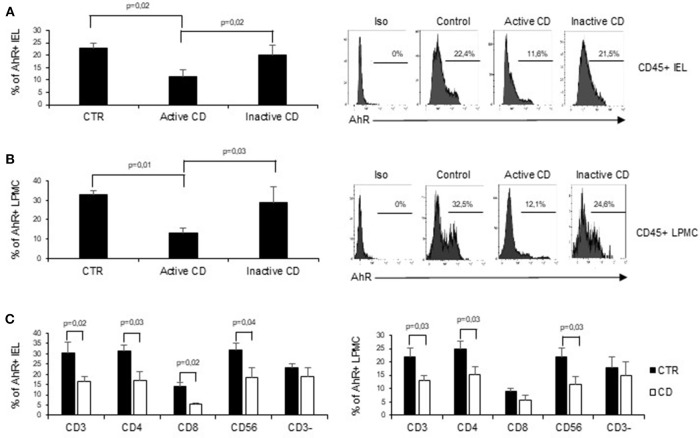
AhR is down-regulated in CD45+ cells isolated from active CD mucosa. **(A)** IEL and LPMC **(B)** were isolated from 6 controls (CTR), 6 active CD patients, and 5 inactive CD patients. Cells were stained with CD45 and AhR antibodies and analyzed by flow-cytometry. Representative histoplots showing the percentage of CD45 positive cells expressing AhR in controls, active CD patients and inactive CD patients (right). Staining with isotype control is also shown (left). **(C)** IEL (left) and LPMC (right) were isolated from 5 controls (CTR) and 5 active CD patients. Cells were stained with CD3, CD4, CD8, CD56, and AhR antibodies and analyzed by flow-cytometry. Histograms show the percentage of CD3+, CD4+, CD8+, CD56+, and CD3− cells expressing AhR. Data are represented as mean ± SEM.

To assess whether CD-related inflammation is characterized by an altered expression of AhR, we initially evaluated AhR RNA transcripts in mucosal biopsies taken from active CD patients, inactive CD patients and controls. AhR RNA expression was significantly reduced in the duodenal mucosa of active CD patients as compared to inactive CD patients and controls, while there was no significant difference between inactive CD and controls (Figure 1A). By immunohistochemistry, we then showed that AhR-positive cells were abundant in duodenal sections taken from controls, while active CD patients displayed a reduced AhR protein expression especially in the lamina propria compartment (Figure 1B). Flow-cytometric analysis confirmed these data showing that the percentage of CD45+ cells expressing AhR was significantly lower in LPMC and IEL isolated from active CD compared to inactive CD patients and controls (Figures 2A,B).

In subsequent experiments, to better understand the specific contribution of AhR in different cell subsets in the context of CD immune response, we analyzed AhR expression in CD3+, CD56+, and CD3− cells. Interestingly the reduction of AhR protein expression was restricted to CD3+T cells and CD56+ natural killer cells. In active CD the percentage of AhR-expressing CD4+ or CD8+ IEL was reduced as compared to controls, while in the lamina propria compartments only AhR-expressing CD4+LPMC were significantly diminished as compared to controls (Figure 2C). On the other end, CD3− cells, isolated from both the lamina propria and the intraepithelial compartment, showed a comparable expression of AhR in the three groups (Figure 2C).

Collectively these findings indicate that CD-associated inflammation is marked by reduced expression of AhR mainly in mucosal T cells and natural killer cells.

### Ficz Reduces the Expression of Inflammatory Cytokines in Celiac Disease Mucosa

**Figure 3:**
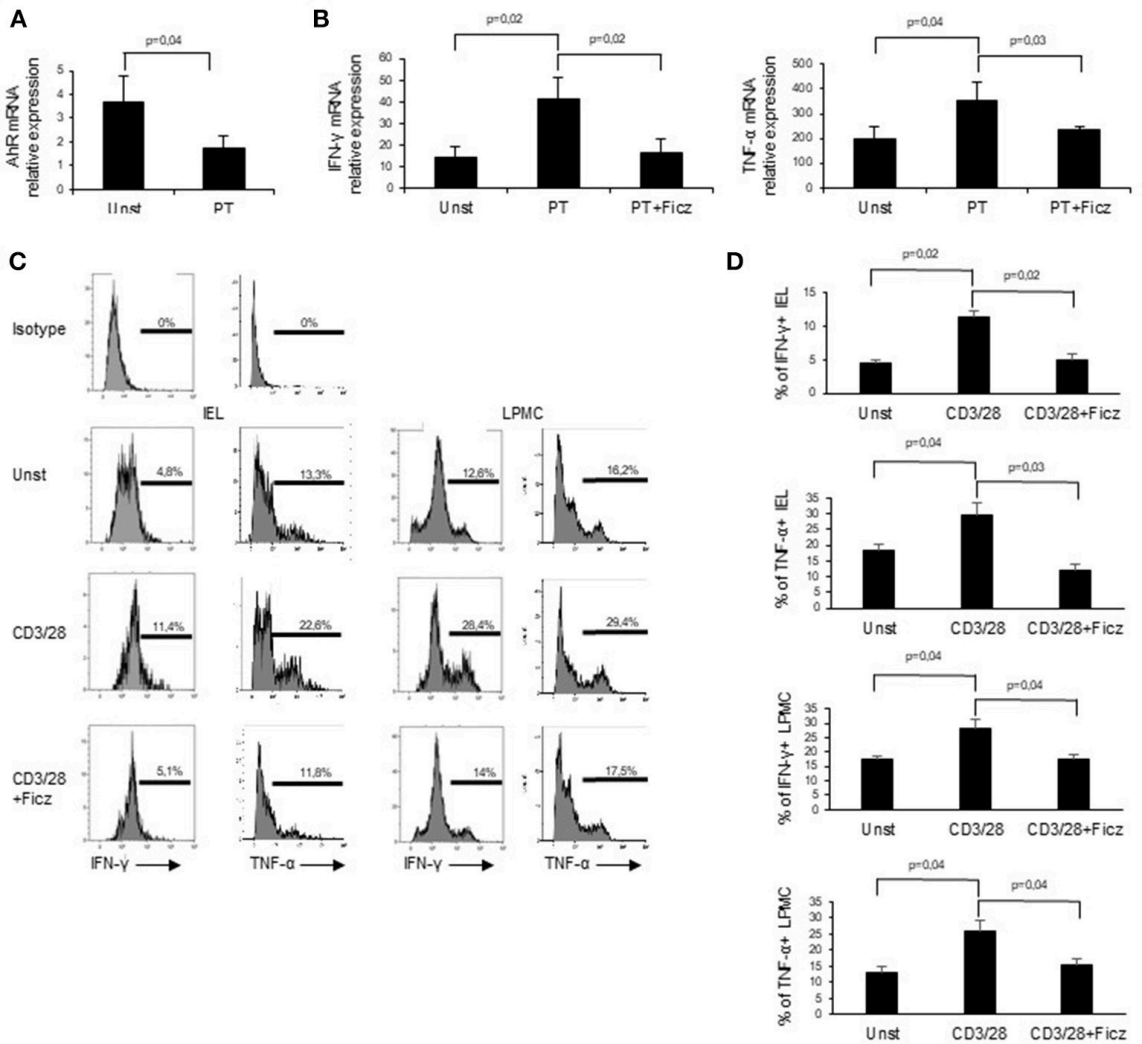
Challenge of inactive CD biopsy specimens with a peptic-tryptic digest of gliadin (PT) results in reduced expression of AhR. **(A)** Duodenal biopsies of 6 inactive CD patients were left untreated (Unst) or stimulated with PT for 24 h and AhR expression was evaluated by real-time PCR and normalized to β-actin. Data are presented as mean ± SEM of 6 separate experiments. **(B)** IFN-γ (left) and TNF-α (right) RNA expression was evaluated by real time PCR in duodenal biopsies of inactive CD patients left untreated (Unst) or stimulated with PT, with or without Ficz for 24 h. Data indicate mean ± SEM of 5 separate experiments. The AhR ligand Ficz reduces the expression of inflammatory cytokines in CD. **(C,D)** IEL and LMPC were isolated from 6 active CD patients and treated with anti-CD3/CD28 and Ficz for 48 h. Representative histoplots showing IFN-γ and TNF-α expression in IEL analyzed by flow-cytometry. Percentage of IEL (**A**, left histograms) and LPMC (**A**, right histograms) (**B**, left graphs) and LPMC (**B**, right graphs) isolated from 6 active CD patients and treated with anti-CD3/CD28 with or without Ficz were stained with CD45, IFN-γ and TNF-α antibodies and analyzed by flow-cytometry. Data are shown as mean ± SEM of n separate experiments.

Next, we examined whether PT influences AhR mucosal expression. To this end, we collected duodenal biopsies taken from patients with inactive CD on a gluten-free diet and treated them *in vitro* with PT. PT administration led to a significant down-regulation of AhR transcripts (Figure 3A). In subsequent experiments, we analyzed the effect of AhR activation on the expression of pro-inflammatory cytokines, such as IFN-γ and TNF-α, which have a role in CD-related inflammation. Duodenal biopsies collected from inactive CD patients and treated with PT showed an increased expression of IFN-γ and TNF-α transcripts, while co-incubation with Ficz completely reverted the effect of PT (Figure 3B). To confirm the protective effect of AhR activation, we collected LPMC and IEL isolated from the inflamed mucosa of active CD patients and treated them with anti-CD3/CD28 in the absence or presence of Ficz. Treatment with Ficz reduced anti-CD3/CD28-driven IFN-γ and TNF-α expression both in LPMC and IEL (Figures 3C,D). Collectively these data suggest that defective AhR mucosal expression can contribute to amplify cytokine expression in active CD.

### AhR Activation Reduces the Expression of Cytotoxic Factors in Active Celiac Disease

**Figure 4:**
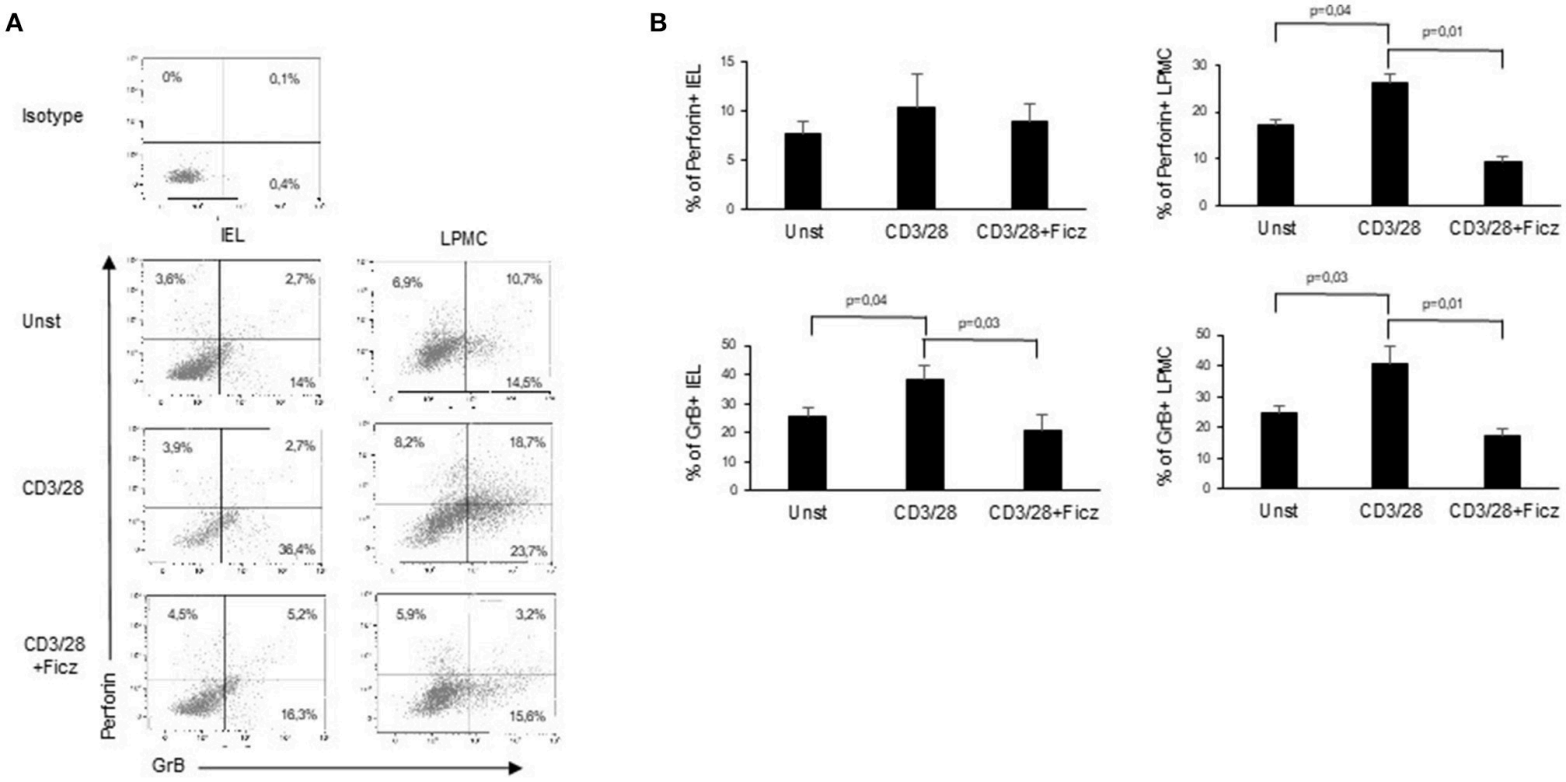
Treatment with Ficz reduces the expression of cytotoxic factors in CD. IEL and LPMC isolated from 6 active CD patients and treated with anti-CD3/CD28 with or without Ficz for 48 h were stained with CD45, perforin and granzyme B antibodies and analyzed by flow-cytometry. **(A)** Representative dot plots showing CD45 positive IEL and LPMC expressing perforin and granzyme. Representative staining with isotype control is also shown. **(B)** Percentage of CD45 positive IEL and LPMC expressing perforin and granzyme B. Data are shown as mean ± SEM of 6 separate experiments.

In active CD, the production of inflammatory cytokines triggers the activation of cytotoxic factors, which ultimately determine villous atrophy, the main histological feature of CD. Since cytotoxic cells are supposed to play a role in the CD-associated intestinal damage (8), we investigated whether AhR controls the expression of cytotoxic factors. For this purpose, LPMC and IEL of active CD patients were treated with Ficz and analyzed for the expression of cytotoxic factors. Ficz reduced the percentage of LPMC and IEL expressing perforin and granzyme B (Figures 4A,B). These data support the anti-inflammatory role of AhR in CD mucosa.

### AhR Activation Prevents Poly I:C-Induced Intestinal Damage

**Figure 5:**
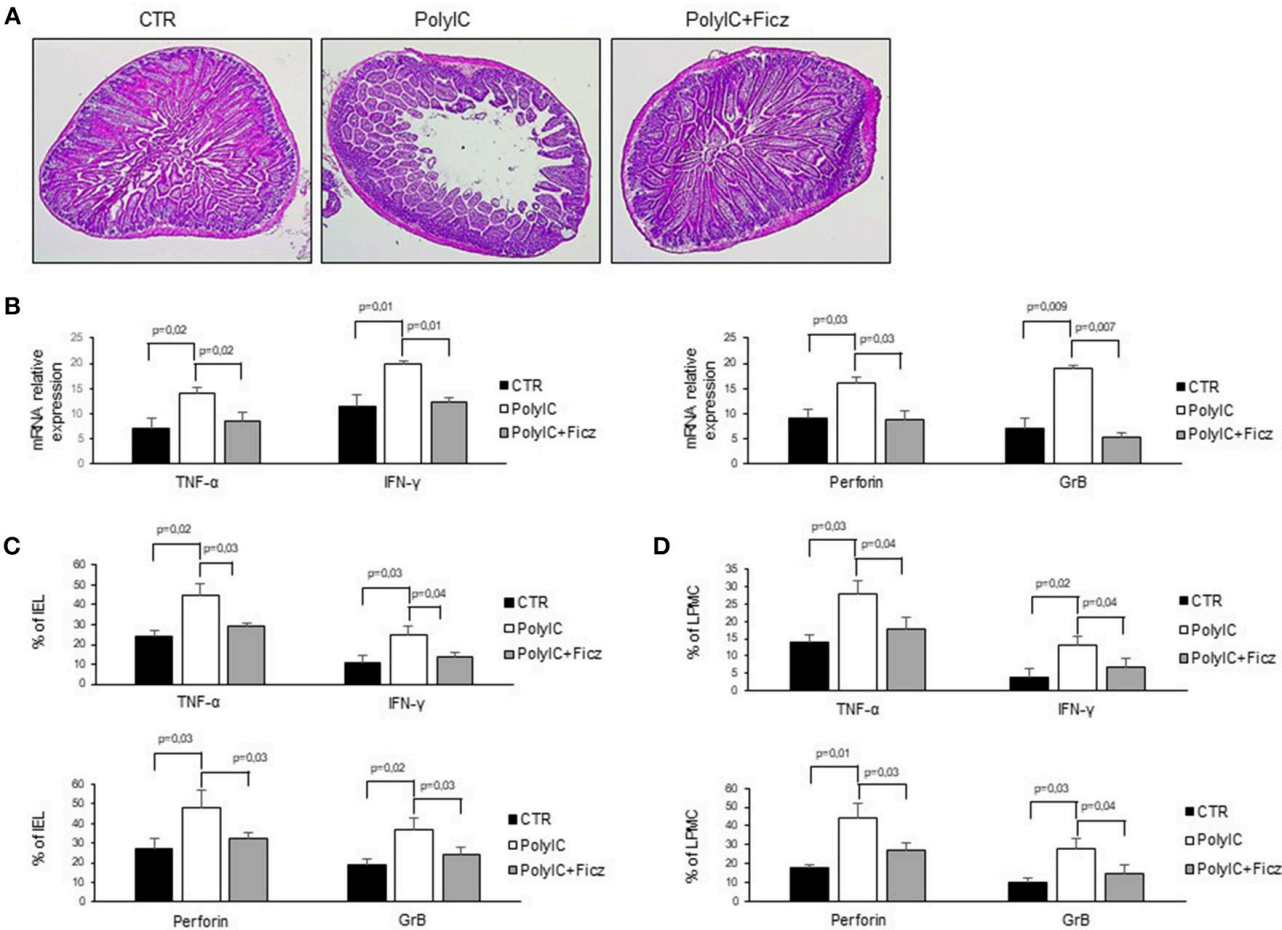
AhR activation ameliorates poly I:C-induced villous atrophy. **(A)** Representative H&E-stained small intestinal sections of mice left untreated (CTR), receiving poly I:C (poly I:C) or treated with Ficz (poly I:C+Ficz). **(B)** TNF-α, IFN-γ (left), perforin and granzyme B (right) RNA transcripts were analyzed by real-time PCR and normalized to β-actin in the small intestine of control mice (CTR), mice treated with poly I:C (poly I:C), and mice receiving poly I:C and treated with Ficz (poly I:C+Ficz). **(C,D)** IEL and LPMC were isolated from the small intestine of control mice (CTR), mice treated with poly I:C (poly I:C) and receiving poly I:C and treated with Ficz (poly I:C+Ficz), and stained with CD45, TNF-α, IFN-γ, perforin, and granzyme B analyzed by flow cytometry. All data indicate mean ± SEM of 3 separated experiments in which at least 4 mice per group were considered.

In mice intra-peritoneal injection of poly I:C triggers activation of intestinal mucosal immune cells thereby leading to small intestinal epithelial lesions (24–26). Therefore, we hypothesized that AhR activation can attenuate poly I:C-driven intestinal lesions. To this end, mice were treated with poly I:C with or without Ficz. As expected, treatment of mice with poly I:C induced intestinal damage characterized by flattening of small intestinal villi (Figure 5A) and significantly increased TNF-α, IFN-γ, perforin, and granzyme B RNA expression as compared to control mice (Figure 5B). Ficz administration prevented poly I:C-driven intestinal damage and TNF-α, IFN-γ, perforin, and granzyme B RNA induction (Figure 5B). Accordingly, Ficz significantly reduced the fractions of TNF-α, IFN-γ, perforin, and granzyme B-expressing LPMC and IEL induced by poly I:C (Figures 5C,D).

**Figure 6:**
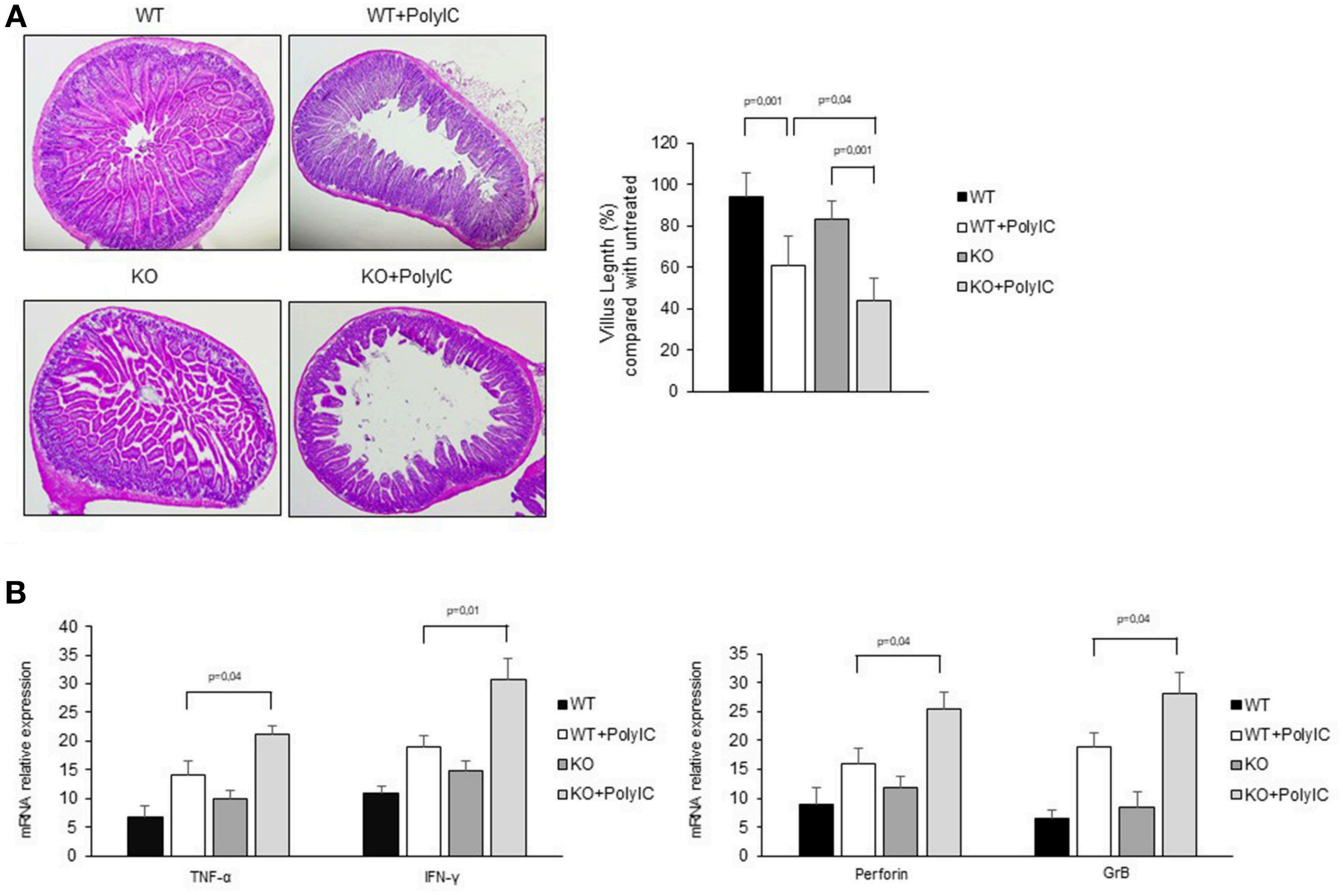
Following poly I:C administration, AhR knock-out mice produce more inflammatory factors than wild-type mice. **(A)** Representative H&E-stained small intestinal sections of wild-type (WT) and AhR knock-out (KO) mice left untreated (left) or receiving poly I:C (poly I:C; right). **(B)** Morphometric analysis of villous length between the different groups as an index of small intestinal lesions. All data indicate mean ± SD of 3 separated experiments in which at least 4 mice per group were considered. **(C)** TNF-α, IFN-γ (left), perforin and granzyme B (right) RNA transcripts were analyzed by real-time PCR and normalized to β-actin in the small intestine of WT and AhR KO mice either left untreated or treated with poly I:C (poly I:C). All data indicate mean ± SEM of 3 separated experiments in which at least 4 mice per group were considered.

To further confirm the anti-inflammatory role of AhR in the intestinal mucosa, poly I:C was administered by intra-peritoneal injection to WT and AhR KO mice and cytokines and cytotoxic factors were analyzed by real-time PCR. Both WT and AhR KO mice showed marked intestinal lesions, however, this damage was significantly more evident in AhR KO mice (Figures 6A,B). As shown in Figures 6C,D, the RNA transcripts for TNF-α, IFN-γ, perforin, and granzyme B were increased in both WT and AhR KO mice following poly I:C administration but such expression was significantly greater in AhR KO mice than in WT mice (Figures 6C,D). Altogether, these data indicate that AhR triggers anti-inflammatory signals in the gut.

## Discussion

In this study, we examined the expression and functional role of AhR in CD. We initially documented a diminished expression of AhR in the intestinal mucosa of patients with active CD as compared with inactive CD patients and normal controls. Analysis of AhR in distinct mucosal cell types by flow-cytometry revealed a significant reduction in both CD4+ and CD8+ T cells infiltrating the epithelial layer while the protein was reduced only in CD4+ LPMC, clearly suggesting a cell-specific regulation of AhR in distinct mucosal compartments. Unfortunately, the number of cells purified from duodenal biopsy samples was not sufficient to further assess the expression of AhR in additional cell types (e.g., tissue-resident memory T cells, CD4+ CD8αα+ double-positive T cells), which have been associated with AhR function in other systems (27).

The mechanisms that regulate AhR expression in CD mucosa remain to be ascertained. *Ex vivo* organ culture experiments revealed that treatment of duodenal biopsies taken from inactive CD patients with PT reduced AhR expression. Although these data would seem to suggest a direct and negative effect of dietary gluten on AhR, we have previously documented a diminished expression of AhR in Crohn's disease, another chronic inflammatory disorder of the gut (22), which is not driven by gluten. Therefore, it is conceivable that AhR can be down-regulated by either inflammatory molecules (e.g., IL-15 and Th1 cytokines), which are over-produced in both CD and Crohn's disease, or factors that are differently synthesized in the two disorders. The fact that AhR content is reduced in mucosal cells (i.e., T cells), which are supposed to make a robust contribution to CD pathogenesis (1), and AhR triggers counter-regulatory effects in other systems (22, 28, 29), prompted us to explore the possibility that activation of AhR with exogenous ligands could dampen CD-associated inflammatory pathways. To this end, CD IEL and LPMC were activated with anti-CD3/CD28 antibodies in the presence or absence of Ficz. Ficz reduced the percentages of IFN-γ and TNF-α-expressing IEL and LPMC. Consistently, Ficz abolished the induction of both cytokines in duodenal biopsies taken from inactive CD patients exposed to PT. Overall, these results are in agreement with data of previous studies showing that AhR activation reduces the production of inflammatory cytokines in mucosal cells of Crohn's disease patients and murine models of intestinal inflammation (22, 30). Taken together, these observations indicate that AhR delivers negative signals on T cell activation in the human gut. The factors/mechanisms underlying such a regulatory effect of AhR on T cells remain to be determined. One possibility is that AhR can directly inhibit the transcription of inflammatory genes. Indeed, in other experimental models, it has previously been shown that AhR interacts with STAT1 on the IL-6 promoter and suppresses LPS-induced activation of IL-6 expression by inhibiting the transcriptional activity of NF-κB in murine macrophages (31). It is also possible that AhR can favor the differentiation/activation of regulatory T cells or synthesis of immunesuppressive cytokines (e.g., IL-10), which in turn suppress effector T cell responses. Indeed, Goettel et al. showed that activation of AhR with a non-toxic agonist increased IL10-secreting human Tregs and ameliorated colitis in humanized mice (32).

Our data also indicate that activation of AhR reduces the release of cytotoxic products, such as granzyme B and perforin in IEL and LPMC isolated from active CD, thus confirming the regulatory effect of AhR on cytotoxic cell proliferation and functions in the gut (33). To further confirm the anti-inflammatory role of AhR in the gut, we used a model of small intestinal lesions induced in mice by poly I:C intraperitoneal injection (25). Our data indicated that Ficz protected mice from poly I:C-induced intestinal damage and reduced levels of inflammatory cytokines and cytotoxic factors. We are aware that the poly I:C-induced enteropathy does not recapitulate the major immunological/morphological features of CD. However, in this model, the epithelial lesions are supposed to be driven by inflammatory pathways (i.e., TNF signaling, perforin, and granzyme activity), which are activated in CD.

In conclusion, our findings suggest that defective AhR signaling could contribute to amplify detrimental immune signals in CD mucosa.

## Author Contributions

IvM conceived and designed the experiments. VD, IrM, DD, AD, FL, and IvM performed the experiments. IvM and GM analyzed the data. RD and OP contributed reagents, materials, analysis tools. VD, IrM, IvM, and GM wrote the paper.

### Conflict of Interest Statement

GM has served as an advisory board member for ABBVIE. The remaining authors declare that the research was conducted in the absence of any commercial or financial relationships that could be construed as a potential conflict of interest.
